# Predictive screening of M1 and M2 macrophages reveals the immunomodulatory effectiveness of post spinal cord injury azithromycin treatment

**DOI:** 10.1038/srep40144

**Published:** 2017-01-06

**Authors:** John C. Gensel, Timothy J. Kopper, Bei Zhang, Michael B. Orr, William M. Bailey

**Affiliations:** 1 Spinal Cord and Brain Injury Research Center, Department of Physiology, College of Medicine University of Kentucky Lexington, Kentucky 40536.

## Abstract

Spinal cord injury (SCI) triggers a heterogeneous macrophage response that when experimentally polarized toward alternative forms of activation (M2 macrophages) promotes tissue and functional recovery. There are limited pharmacological therapies that can drive this reparative inflammatory state. In the current study, we used *in vitro* systems to comprehensively defined markers of macrophages with known pathological (M1) and reparative (M2) properties in SCI. We then used these markers to objectively define the macrophage activation states after SCI in response to delayed azithromycin treatment. Mice were subjected to moderate-severe thoracic contusion SCI. Azithromycin or vehicle was administered beginning 30 minutes post-SCI and then daily for 3 or 7 days post injury (dpi). We detected a dose-dependent polarization toward purportedly protective M2 macrophages with daily AZM treatment. Specifically, AZM doses of 10, 40, or 160 mg/kg decreased M1 macrophage gene expression at 3 dpi while the lowest (10 mg/kg) and highest (160 mg/kg) doses increased M2 macrophage gene expression at 7 dpi. Azithromycin has documented immunomodulatory properties and is commonly prescribed to treat infections in SCI individuals. This work demonstrates the utility of objective, comprehensive macrophage gene profiling for evaluating immunomodulatory SCI therapies and highlights azithromycin as a promising agent for SCI treatment.

Spinal cord injury (SCI) activates CNS macrophages consisting of endogenous microglia and recruited monocyte-derived macrophages. These cells have pathological properties and contribute to neurodegeneration and tissue loss subsequent to the initial mechanical trauma. Activated macrophages also have the potential to promote tissue remodeling and axon regeneration^1^,^2^,^3^ and there is growing evidence that transgenic, viral, or transplantation-mediated alterations in macrophage phenotype in SCI can be therapeutic^4^. The development of clinically viable, pharmacological interventions that harness the reparative potential of macrophages responding to SCI, however, remains a challenge facing the SCI community. To date, only one pharmacological agent, methylprednisolone, is approved to treat SCI and its use is controversial. Increasingly, well-tolerated, pharmacological agents with immunomodulatory properties are being sought as therapies for SCI.

Azithromycin (AZM) is a semisynthetic derivative of the macrolide antibiotic erythromycin with significant immunomodulatory actions independent of is antibacterial properties^5^,^6^. Specifically, AZM treatment in rodent and human inflammatory conditions is associated with improved outcomes coincident with alterations in macrophage phenotype and function^7^,^8^,^9^,^10^,^11^. Further, AZM alters macrophage phenotype in experimental stroke^12^,^13^ and we recently discovered that AZM alters macrophage phenotype and improves recovery when treatment is initiated prior to SCI^14^. AZM is commonly used to treat infections in SCI individuals^15^,^16^ and is therefore safe for this patient population, however, its effectiveness when delivered post-SCI is unknown. The goal of the current work was to test the immunomodulatory potential and dose-response of AZM treatment initiated after experimental SCI.

Work done primarily through *in vitro* systems has identified macrophage phenotypes with pathological and reparative potential in SCI. Specifically, IFNγ + LPS stimulated, M1 macrophages are neurotoxic and cause axon dieback while IL-4 stimulated, M2 macrophages promote axon growth and remyelination without inducing cell death^3^,^17^,^18^,^19^,^20^. Therefore, markers of M1 or M2 macrophages are routinely used to evaluate the efficacy of SCI therapies. There is a growing appreciation, however, that macrophages display a range of phenotypes *in vivo* and that, in solitary, M1 or M2 markers may not sufficiently capture the inflammatory status of the injured CNS^21^,^22^,^23^. Instead, knowing the relative proportion of a combination of M1 or M2 markers and transcription factors is favored by macrophage biologists for determining the phenotypic state of macrophages in response to different pathological or treatment conditions^24^,^25^,^26^. In the current study, we used small-scale gene array to identify a comprehensive set of genetic markers to identify macrophages with published pathological (M1) and reparative properties (M2) for SCI. We then used these markers to objectively determine the dose-response and efficacy of post-SCI AZM treatment.

## Results

**Figure 1:**
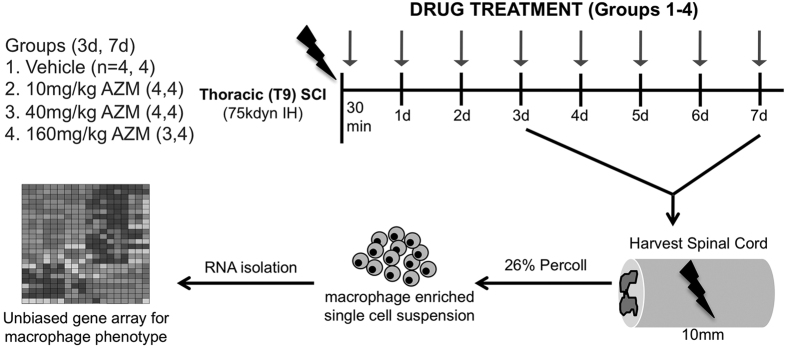
Diagram of experimental design. Adult (4 months old) female mice were subjected to moderate-severe thoracic contusion SCI. AZM (azithromycin) or vehicle was administered beginning 30 minutes post-SCI and then daily throughout the survival period (3 or 7 dpi). Macrophages were then isolated from the injured spinal cord and subject to gene array for phenotyping. An additional set of animals (not shown in figure) was use for histological analysis (n = 8/group). IH = Infinite Horizons Impactor.

Previously we observed that AZM treatment, begun prior to SCI, alters macrophage activation and improves tissue repair and functional recovery^14^. The purpose of this study was to determine if AZM treatment, initiated after SCI, alters the macrophage response to injury. Figure 1 shows the experimental design in which adult (4 months old) C57BL/6 mice received SCI. Vehicle or drug treatment (10, 40 or 160 mg/kg AZM via oral gavage) was given 30 minutes post-SCI then daily until tissue collection at 3 or 7 days post injury (dpi). Small-scale gene array was then used to determine the phenotype of macrophages isolated from the injured spinal cord.

**Figure 2:**
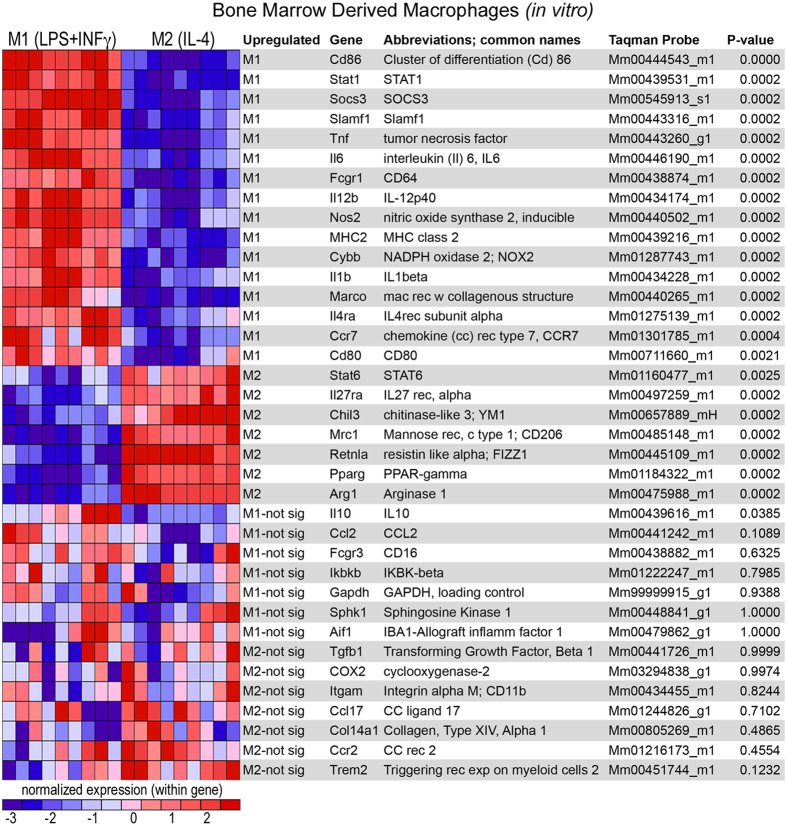
Distinct genes are upregulated by M1 and M2 macrophages *in vitro*. Gene expression of bone marrow-derived macrophages (BMDM) stimulated to M1 (IFNγ + LPS) or M2 (IL-4). RNA was isolated 6 hrs after stimulation then analyzed using a customized TLDA array gene card. The results of the Comparative Marker Selection suite (Genepattern) revealed significant upregulation (p < 0.01 FWER) of 16 genes in M1 BMDMs and 7 genes in M2 BMDMs. Genes are listed in rank order by their specificity to each phenotype (top to bottom for M1 and bottom to top for M2). Results are comprehensive from three independent biological experiments each with three replicates for each stimulation. Abbreviations: exp (expressed), Fcgr1 (Fc receptor, IgG, low affinity), IKBK (inhibitor of nuclear factor kappa-B kinase), MHC (major histocompatibility complex), mac (macrophage), NADPH nicotinamide adenine dinucleotide phosphate), PPAR (Peroxisome proliferator-activated receptor), rec (receptor), Slamf1 (signaling lymphocytic activation molecule family member), STAT (Signal transducer and activator of transcription), SOCS (suppressor of cytokine signaling).

To objectively define markers specific to M1 or M2 macrophages, we performed gene expression profiling of unstimulated (M0), IFNγ + LPS (M1), or IL-4 (M2) stimulated bone marrow-derived macrophages (BMDM)^3^. We used the Comparative Marker Selection suite (GenePattern) to identify genes significantly upregulated by either M1 or M2 BMDMs^24^,^27^. After controlling for family-wise error, 16 genes were significantly upregulated (using a conservative p < 0.01 FWER cutoff for significance) by M1 BMDMs and 7 genes were upregulated by M2 BMDMs (Fig. 2). Since we previously observed that the response of BMDMs *in vitro* is predictive of SCI macrophages *in vivo*^1^,^28^, we used the genes presented in Fig. 2 as an objective, unbiased, a priori means of phenotyping M1 and M2 macrophages in SCI.

**Figure 3:**
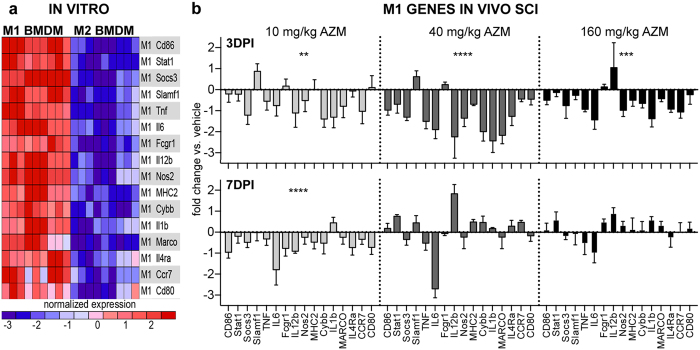
Post-SCI AZM treatment significantly decreases M1 gene expression in macrophages. (**a**) The 16 genes significantly upregulated by M1 bone marrow-derived macrophages *in vitro* (data reproduced from Fig. 2). These genes were used a priori to examine the M1 response to SCI treatment. (**b**) Gene expression of Percoll-enriched macrophages isolated from the injured spinal cord. Adult (4 month old) female mice received a moderate-severe thoracic contusion SCI and AZM or vehicle treatment was initiated 30 mins post-SCI then daily for 3 or 7 days. Gene expression is normalized to same-day vehicle control (control = 0). Regardless of dose, AZM significantly reduces M1 gene expression compared to vehicle at 3 days post injury (dpi). Note the almost homogenous down regulation of M1 genes (14 of 16, 87.5%) for 40 and 160 mg/kg treatment groups at 3 dpi. The 10 mg/kg dose also decreases M1 gene expression at 7 dpi. **, ***, ****p < 0.01, 0.001, or 0.0001 vs. vehicle (main effect of treatment), n = 3–4, mean +/− SEM.

Next, we used the genes identified in Fig. 2 to determine the dose-dependent effect of delayed AZM treatment on SCI macrophage phenotypes. AZM or vehicle was delivered 30 mins after SCI then daily until 3 or 7 days post injury (dpi) when macrophages were isolated from the injured spinal cord for phenotype analysis. These time points bracket the onset and peak of SCI macrophage activation and include a dynamic period of M1 and M2 activation states^3^,^20^,^29^. For each time point, gene expression was first normalized to the same-day vehicle control. There were significant main effects of treatment for collective M1 gene expression at both 3 (F3, 174 = 22.18, p < 0.0001, n = 3–4) and 7 dpi (F3, 192 = 9.95, p < 0.0001, n = 4) (Fig. 3). For 3 dpi, M1 gene expression was significantly reduced for all treatment groups compared to vehicle (p < 0.005 main effect vs. vehicle for each dose) (Fig. 3). These main effects were driven in large part by downregulation of Socs3, IL6, IL12b, IL1β, iNOS (NOS2), Nox2 (cybb) and to a lesser extent TNF-α (Fig. 3). At 7 dpi, M1 gene expression was significantly reduced compared to vehicle for the 10 mg/kg dose (p < 0.0001) (Fig. 3). This was in part due to a large decrease in IL6 although 94% of the M1 genes (15/16) were decreased in this group relative to vehicle (Fig. 3).

**Figure 4:**
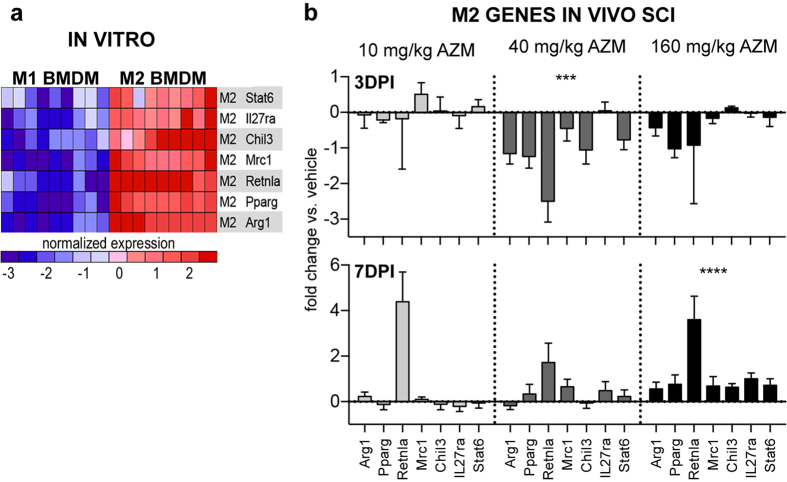
Post-SCI AZM treatment significantly increases M2 gene expression in macrophages. (**a**) The 7 genes significantly upregulated by M2 bone marrow-derived macrophages *in vitro* (data reproduced from Fig. 2). These genes were used a priori to examine the M2 response to SCI treatment. (**b**) Gene expression of Percoll-enriched macrophages isolated from the injured spinal cord. Adult (4 month old) female mice received a moderate-severe thoracic contusion SCI and AZM or vehicle treatment was initiated 30 mins post-SCI then daily for 3 or 7 days. Gene expression is normalized to same-day vehicle control (control = 0). At 3 dpi with the 40 mg/kg dose, M2 genes were significantly suppressed. AZM significantly upregulates M2 gene expression at 7 dpi with the 160 mg/kg dose. Note that all the M2 genes (7/7, 100%) are upregulated vs. vehicle with the 160 mg/kg at 7 dpi. There was a trend for increased M2 expression with the 10 mg/kg dose at 7 dpi. ***, ****p < 0.001, or 0.0001 vs. vehicle (main effect of treatment), n = 3–4, mean +/− SEM.

**Figure 5:**
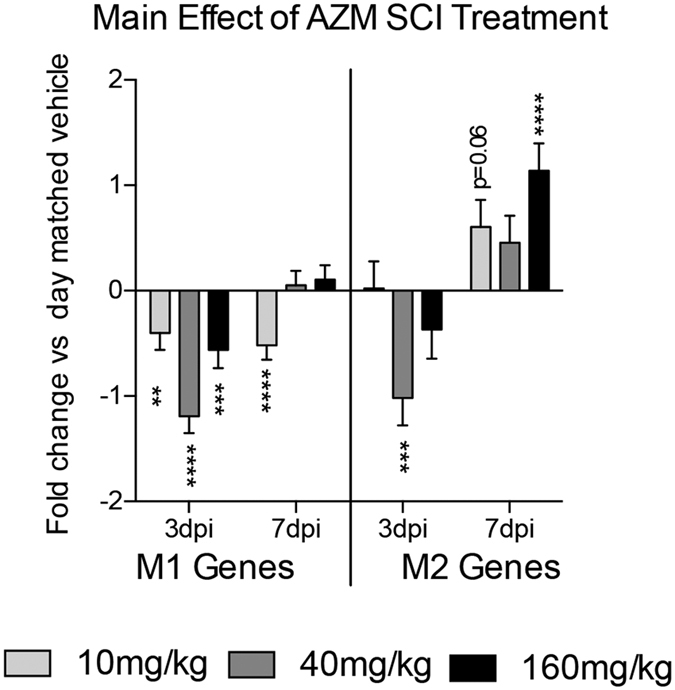
Patterns of M1 and M2 gene expression over time reveal a shift in macrophage phenotype with AZM treatment. Mean expression values of Percoll-enriched macrophages isolated from the injured spinal cord. Adult (4 month old) female mice received a moderate-severe thoracic contusion SCI and AZM or vehicle treatment was initiated 30 mins post-SCI then daily for 3 or 7 days. Values are the mean of all M1 or M2 genes from Figs 3 and 4. Gene expression is normalized to same-day vehicle control (control = 0). Both the 10 mg/kg and 160 mg/kg AZM doses significantly decreased M1 gene expression at 3 dpi with a subsequent increase in M2 expression at 7 dpi. Similar non-significant trends were observable with the 40 mg/kg dose; however, M2 gene expression was significantly suppressed in this group at 3 dpi. **, ***, ****p < 0.01, 0.001, or 0.0001 vs. vehicle (main effect of treatment), n = 3–4, mean +/− SEM.

There was a significant treatment effect for collective M2 gene expression at both 3 (F3, 77 = 7.08, p = 0.0003, n = 3–4) and 7 dpi (F3, 84 = 6.62, p = 0.0005, n = 4) (Figs 4 and 5). At 3 dpi, M2 gene expression was significantly reduced for the 40 mg/kg treatment group (main effect vs. vehicle, p = 0.0005) due largely to decreases in Arginase 1 (Arg1), peroxisome proliferator-activated receptor- γ (Ppar-γ), Fizz1 (Retnla), and YM-1 (Chil3) although 86% of the M2 genes were downregulated in this treatment group (6/7) (Figs 4 and 5). At 7 dpi there was a significant increase in M2 gene expression with 160 mg/kg treatment (main effect vs. vehicle, p < 0.0001) (Figs 4 and 5A) with 100% of M2 genes (7/7) upregulated compared to vehicle and Fizz1 (Retnla) showing the greatest treatment effect (Fig. 4). There was a trend for increased M2 gene expression in the 10 mg/kg group at 7 dpi (p = 0.06) driven largely an increase in Fizz1 (Retnla) expression.

**Figure 6:**
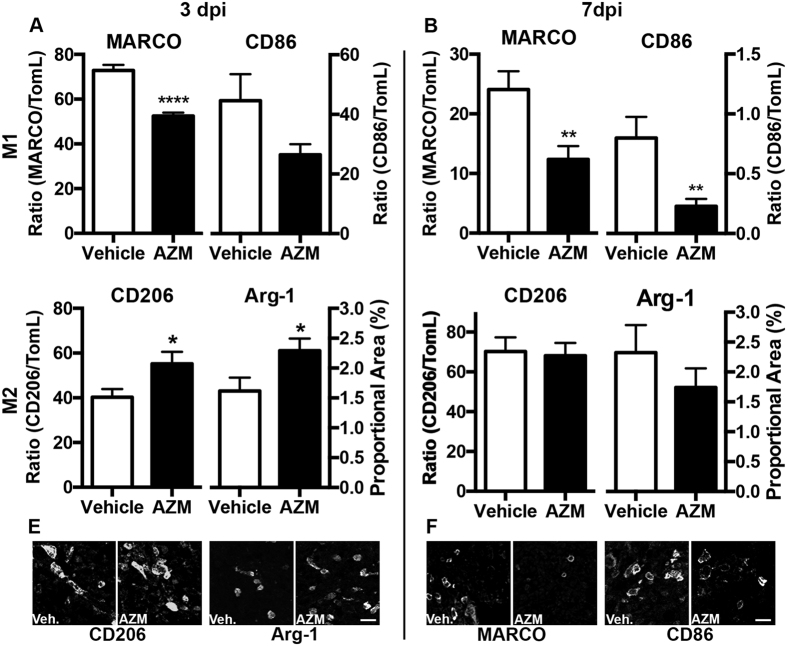
Post-SCI AZM treatment significantly decreases M1 protein expression and increases M2 gene expression in macrophages. Immunohistochemical double-labeling of M1 (MARCO, CD86) or M2 (CD206, Arg-1) markers with macrophages (Tomato lectin+) at the lesion epicenter at 3 and 7 dpi. Adult (4 month old) female mice received a moderate-severe thoracic contusion SCI and AZM (160 mg/kg) or vehicle treatment was initiated 30 mins post-SCI then daily for 3 or 7 days. (**A,B**) AZM significantly decreases the proportion of macrophages positive for the M1 markers MARCO and CD86 relative to vehicle. (**C**) AZM significantly increases the proportion of macrophages positive for the M2 marker CD206 and Arg-1 at 3 dpi but not 7 dpi relative to vehicle. (**E**) Representative images of CD206 and Arg-1 labeling in the lesion epicenter at 3 dpi. (**F**) Representative images of MARCO and CD86 labeling in the lesion epicenter at 7 dpi. *p  <  0.05, **p  <  0.01, ****p  <  0.0001 vs. vehicle. n = 7–8, mean ± SEM. Scale bar = 20 μm.

To determine if gene expression was indicative of changes at the protein level, we examined histological expression of M1 and M2 markers on spinal cord tissue sections. Specifically, we examined the markers with highest rank order specificity for M1 (CD86) and M2 (Arginase-1) macrophages as revealed from *in vitro* gene analysis (Fig. 2) and two commonly used M1 and M2 markers (MARCO and CD206 respectively)^22^ in spinal cord sections from animals treated with AZM (160 mg/kg) or vehicle (treatment initiated 30 mins post SCI then daily) at 3 or 7 dpi. Phenotypic markers co-localized almost exclusively with IBA-1-labeled macrophages (see Supplemental Fig. S1). Consistent with gene expression data, AZM treatment significantly decreased macrophage M1 labeling and increased macrophage M2 labeling in the spinal cord after SCI (Fig. 6).

Collectively, these data show that post SCI AZM treatment alters macrophage phenotype in a dose-dependent manner. The 160 mg/kg dose decreases M1 gene and protein expression and increases M2 gene and protein expression relative to vehicle treatment. The 10 and 40 mg/kg treatment doses have similar immunomodulatory effects on M1 gene expression but less effectively increase M2 gene expression (Fig. 5).

## Discussion

In a recent editorial, Siamon Gordon and Fernando Martinez, two scientists instrumental in the establishment of the M1:M2 paradigm, called for comprehensive classification systems and iterative comparisons across *in vitro* and *in vivo* systems to better identify therapeutic targets for disease^21^. In the current study, we used *in vitro* systems to comprehensively defined markers of M1 and M2 macrophages with known pathological and reparative properties in SCI. We then used these markers to objectively define the macrophage activation states after SCI in response to delayed AZM treatment. The principle finding in this report is that oral administration of this common macrolide antibiotic alters SCI macrophage phenotype when treatment is initiated after injury. AZM was observed to decrease M1 gene expression and increase M2 gene expression after SCI. Specifically, AZM doses ranging from 10–160 mg/kg decreased M1 gene expression in macrophages while both the lowest (10 mg/kg) and highest (160 mg/kg) doses administered increased M2 macrophage gene expression. Previously, we observed that this AZM-mediated shift in polarization following a pretreatment paradigm is associated with improved tissue sparing and functional recovery^14^. Therefore, these findings highlight the potential for AZM to serve as an effective treatment for SCI. Further, this approach demonstrates the utility of objective, comprehensive macrophage gene profiling for evaluating immunomodulatory SCI therapies.

The M1:M2 paradigm is a useful framework for comparisons across different disease states and between *in vivo* and *in vitro* systems. There is growing appreciation, however, that this bipolar classification system greatly oversimplifies the dynamic and complex macrophage activation at sites of neurotrauma^23^. Indeed, while the results of the current work provide insight into the inflammatory states induced with AZM treatment, it is unlikely that exclusive M1 or M2 cells are being attenuated or boosted by treatment. It is reasonable that the milieu of stimuli present after SCI induces complex, heterogeneous, and mixed M1 and M2 macrophage activation states, potentially even within individual cells as reported for TBI^30^. Nonetheless, we noted almost global suppression of M1 gene expression with AZM treatment at 3 dpi (14 of 16 M1 genes (87.5%)) and subsequent upregulation of all M2 genes at 7 dpi (7/7, 100%) with the highest AZM dose. Therefore, the overall effect of AZM administration may be to modulate macrophage activation in favor of increased expression of genes that promote repair as opposed to selectively inhibit or activate M1 and M2 cells.

Although we examined gene expression from enriched macrophage preparations, and not whole tissue homogenates, it is difficult to discern whether the AZM-mediated shifts in macrophage gene expression are due to the indirect action of AZM on upstream mediators of macrophage activation or direct macrophage-AZM interactions. Extensive *in vitro* data demonstrate that AZM interferes with pro-inflammatory macrophage activation^5^,^14^,^31^,^32^,^33^. Further, non-antibiotic variants of AZM are able to attenuate pro-inflammatory macrophage activation *in vivo* and *in vitro*^34^,^35^. Collectively, these data provide evidence that AZM has the potential to directly modulate macrophage activation independent of its antibiotic capabilities. Nonetheless, AZM treatment is also associated with neutrophil alterations in activity and expression of adhesion molecules *in vivo*^36^. Soon after SCI, neutrophils accumulate in the spinal cord, breakdown and remove cellular debris, and then undergo apoptosis and are subsequently removed by macrophages. Recently, in a model of myocardial infarction, it was reported that spent neutrophils are a potent stimulus that may regulate the phenotypic balance of M1 and M2 macrophage activation states^37^. In the current study, AZM could be interfering with neutrophil activity resulting in indirect changes in macrophage gene expression.

It has also been recently reported that macrolides promote macrophage recruitment to areas of upper respiratory tract infection^38^. Interestingly, in that study, AZM treatment increased expression of CCL2 (or monocyte-chemoattractant protein-1, MCP-1) *in vitro* and macrolide treatment increased CCL2 expression *in vivo*. CCL2 is a ligand for CCR2, a monocyte receptor that facilitates trafficking to areas of spinal and brain injury. AZM-mediated alterations in monocyte-mediated macrophage accumulation in the injured spinal cord could be disrupting the phenotypic balance of M1 and M2 macrophage gene expression observed in the current study.

Azithromycin effectively crosses the blood brain barrier with brain tissue concentrations hundreds of fold higher than serum concentrations^39^. Azithromycin, therefore, has the potential to alter activation of both endogenous microglia and monocyte-derived macrophages at the site of SCI. We are currently developing comprehensive array techniques to definitively phenotype these two different cell populations in response to AZM treatment *in vivo*. This, in combination with recent advances in labeling techniques for AZM^40^, may facilitate further investigations into the cellular populations affected by AZM in neurotrauma. Previously, however, we observed increased expression of M2 markers on activated microglia concomitant with decreased monocyte accumulation with oral AZM administration in SCI^14^. Similarly, intraperitoneal AZM-treatment in a mouse model of stroke decreased inflammatory myeloid cells in the brain and upregulated M2 markers on cells with a phenotypic resemblance to activated microglia^12^. The converging data from these studies raises the possibility that systemic administration of AZM may be effective by altering microglia phenotype. Since AZM is a well-tolerated pharmacotherapy^41^, this has important implications for other neurodegenerative conditions in which pro-inflammatory microglial activation is a purported contributor to pathology.

To become an effective therapy we need a better understanding of the dose efficacy of AZM. Previously, we observed that a relatively high AZM dose (160 mg/kg daily) improves functional recovery from SCI using a pre- and post-treatment paradigm^14^. We have unpublished data demonstrating that this dose improves recovery when administration begins post-SCI (manuscript in preparation). A similarly high dose (150 mg/kg) in a stroke model improved long-term tissue sparing following a single post-reperfusion administration^12^. It should be noted that in the Amantea *et al*. study, a single low of AZM did not effectively increase long term tissue sparing but the ED_50_ for AZM on acute neuroprotection was 0.59 mg/kg. We observed a shift in M1 to M2 gene expression with a repeated low dose of AZM (10 mg/kg). Collectively, these data highlight the potential that repeated, low doses of AZM might be neuroprotective and facilitate neurological recovery.

In summary, the findings of the current study demonstrate that azithromycin treatment, initiated after SCI, alters the macrophage response to injury. This observation is based upon phenotyping of SCI macrophages using an a priori, objective gene-array approach to identify purportedly reparative and pathological macrophages. Azithromycin is a safe, commonly prescribed antibiotic for SCI individuals, is effective following oral administration, is inexpensive, and is stable at room temperatures, making this an ideal pharmocotherapy for the treatment of SCI.

## Materials and Methods

### Animals

Fifty-seven 4-month-old female C57BL/6 mice were used to generate the data for this study (Jackson Laboratory). Fifty-four animals received SCI and one animal died post surgery (SCI N = 31 for gene expression; N = 32 for histology). Three animals were used to generate bone marrow-derived macrophage (BMDMs N = 3). Animals were housed in IVC cages with ad libitum access to food and water. All procedures were performed in accordance with the guidelines and protocols of the Office of Research Integrity and with approval of the Institutional Animal Care and Use Committee at the University of Kentucky.

### Spinal cord injury

Animals were anesthetized with ketamine (100 mg/kg) and xylazine (10 mg/kg) (i.p.). After a T9 laminectomy, a moderate-severe thoracic T9 contusion SCI was produced using the Infinite Horizon (IH) injury device (75-kdyn displacement; Precision Systems and Instrumentation)^14^,^42^. A priori criteria were set to exclude animals with abnormalities in the force vs. time curve generated by the IH device at the time of SCI as previously established^43^. These abnormalities are indicative of bone hits or instability in the spinal cord at the time of injury and one animal was excluded in the current study (final N for histology = 31).

Muscle and skin incisions were closed after injury using appropriate monofilament sutures. After surgery, all animals received subcutaneous buprenorphine-SR (1 mg/kg; Zoopharm Pharmacy, Laramie, WY) for pain, preemptive antibiotic (enrofloxacin 5 mg/kg, Norbrook, Lenexa, Kansas), and 2 ml of saline and then were housed overnight in cages warmed by placing them on a 37-degree water pad. Animals continued to receive subcutaneous saline (1 ml) and enrofloxacin for 5 days post-SCI. Food and water intake and the incision site were monitored throughout the course of the study. Manual bladder expression was performed twice daily on SCI mice.

### Drug delivery

After SCI, mice received 10, 40, or 160 mg/kg of Azithromycin (generated by crushing Zithromax tablets and suspending in 1% methylcellulose) or vehicle (1% methylcellulose) in a 0.1 ml volume via oral gavage beginning 30 minutes post-SCI then daily throughout the duration of the experiment (up to 7 days). Animals were randomly assigned to balanced groups based upon the spinal cord displacement at the time of injury. There were no significant differences in spinal cord displacement among groups (ANOVA p > 0.60) thus ensuring that any group differences detected in outcome measures were not due to group injury severity inequalities.

### Isolation of SCI macrophages

Mice were transcardially perfused with ice-cold diethyl pyrocarbonate-PBS (DEPC-PBS) after a lethal dose of i.p. anesthetic (120 mg/kg ketamine, 10 mg/kg xylazine). Ten millimeters of spinal cord (centered on the injury site) was then rapidly dissected and placed in DEPC-PBS on ice. Tissue was dissociated using a plunger from a 5 ml disposable syringe to push spinal cord tissue into 2 mls of ice-cold DEPC-PBS through a 70 μm nylon mesh cell strainer (BD Falcon 35–2350) and flushed with an additional 8 mls of ice-cold DEPC-PBS. Dissociated cells were pelleted at 600 g for 6 minutes at 4 **°**C. A single cell suspension was prepared by adapting procedures from^44^. Briefly, supernatants were discarded and the pellet re-suspended in 1 ml 26% Percoll (GE Healthcare). An additional 9 ml of 26% Percoll was added then samples were mixed by inversion and spun at 2000 g for 15 minutes. Supernatants were discarded then the pellet was washed in 3 ml ice-cold DEPC-PBS, and spun at 600 g for 6 minutes. Supernatants were discarded and the pellet re-suspended in 1 ml Trizol (Life Technologies) for RNA isolation. Through pilot experiments using flow cytometry we have determined that roughly 80% of the single cells suspension is positive for CD11b. For this reason, Percoll-isolated cells are referred to as “macrophage enriched suspensions”.

### Tissue processing and immunohistochemistry

At 3 or 7 dpi, mice were anesthetized with a lethal dose of ketamine (150 mg/kg) and xylazine (10 mg/kg), then transcardially perfused with cold PBS (0.1 M, pH 7.4), followed by perfusion with cold 4% paraformaldehyde (PFA). Dissected spinal cords (1 cm) were post-fixed for 2 h in 4% PFA, then rinsed and stored in cold phosphate buffer (0.2 M, pH 7.4) overnight at 4 °C. Tissue was cryoprotected in 30% sucrose at 4 °C for 1 week, then rapidly frozen in optimal cutting temperature (OCT) compound (Sakura Finetek USA, Inc.) on dry ice. Tissue was systematically randomized into blocks with equal group distribution in each block to ensure uniformity of staining across groups. Tissue was stored at −80 °C before sectioning. Tissue blocks were cut in serial cross sections (10 μm thick) and mounted onto Colorfrost plus slides (Fisher #12-550-17). For immunohistochemistry, spinal cord sections were warmed for 1 hour at 37 °C and rinsed with 0.1-M PBS. Then, slides were incubated in blocking buffer (0.1-M PBS containing 1% bovine serum albumin, Fisher Scientific, Cat# BP1605), 0.1% Triton X-100 (Sigma-Aldrich, Cat# X-100), 0.1% fish gelatin (Sigma-Aldrich, Cat# G7765), and 5% normal goat or donkey serum (SigmaeAldrich, Cat# G9203; D9663) at room temperature for 1 hour, followed by incubation in blocking buffer containing primary antibodies overnight at 4 °C. Primary antibodies included goat anti-CD206 (1:100 dilution, AF2535, R&D Systems, Minneapolis, MN), goat anti-Arginase 1 (1:100, SC-18354, Santa Cruz Biotechnology, Dallas, TX), rat anti-CD86 (1:100, 553689, BD Biosciences, San Jose, CA), rat anti-MARCO (1:1000, MCA1849, BioRad, Hercules, CA), rabbit anti-Iba 1 (1:1000, Wako Chemicals Cat# 019-19741), chicken anti-GFAP (1:500, Aves Cat# GFAP), and biotinylated Tomato lectin (TomL; 1:1000, L0651; Sigma-Aldrich, St. Louis, MO). On the second day, slides were rinsed in 0.1-M PBS and then incubated with secondary antibodies at room temperature for 1 hour. Secondary antibodies included Donkey anti-goat Alexa Fluor (AF) 488 (1:1000; Life Tech Cat# A11055), Goat anti-rabbit AF546 (1:500, Life Tech Cat# A11010), Biotinylated goat anti-chicken (1:500, Aves Cat# B-1005), Streptavidin AF647 (1:1000 Life Tech Cat# S-21374), and donkey anti-rat AF488 (1:1000 dilution, Life Technologies, Carlsbad, CA). After the last rinse all the slides were coverslipped with Immu-Mount (ThermoFisher Scientific). Full dilution curves were determined for each antibody and specificity confirmed using non-primary controls.

Proportional areas of macrophages were quantified using techniques developed previously^1^,^28^,^45^. Briefly, the density of labeling above background was quantified using threshold-based measures on one tissue section per animal at the lesion epicenter. Maximum projection confocal montages of 3 μm thick z-planes, taken with a 20x objective, were generated with a C2 laser scanning confocal microscope (Nikon Instruments Inc, Melville, NY, USA) and quantified using Metamorph software (Molecular Devices). Due to the diffuse labeling of arginase within macrophages, the proportional area of arginase labeling was determined by averaging three standard sampling areas (0.325 mm^2^) per animal focused on the epicenter lesion penumbra. For surface receptors (CD86, CD206, Marco) the total threshold area was determined for the entire lesion epicenter then normalized to the threshold area for TomL. Previously we established that TomL specifically labels macrophages within the spinal cord lesion epicenter^46^ and there was no effect of AZM treatment on TomL density at either time point (p > 0.05).

### Cell culture

Bone marrow-derived macrophage (BMDM) cultures were obtained from C57BL/6 mice as described previously^1^,^22^. Briefly, bone marrow was flushed from femurs and tibias with DMEM +10% FBS (Dulbecco’s Modified Eagle Medium (DMEM), Life Technologies). Cells were then triturated 3–5 times through an 18-gauge needle then centrifuged at 1200 rpm for 5 minutes. After removing supernatant, red blood cells were lysed in lysis buffer (0.15 M NH_4_Cl, 10 mM KHCO_3_, and 0.1 mM Na_2_EDTA, pH 7.4) then the remaining cells were washed in DMEM +10% FBS and plated at 1 × 10^6^ cells/ml in DMEM supplemented with 1% penicillin/streptomycin, 1% HEPES, 0.001% β-mercaptoethanol, 10% FBS, and 20% supernatant from sL929 cells (a generous gift from Phillip Popovich, The Ohio State University). The sL929 supernatant (which contains macrophage colony-stimulating factor) is needed to promote differentiation of bone marrow cells into macrophages (7–10 d)^47^. Media was changed on days 2, 4, and 6 and then cells re-plated on day 7. Cells were plated at a density of 1 × 10^6^/ml and differentiated into M1 (LPS (50 ng/ml; Invivogen) +IFNγ (20 ng/ml; eBioscience, San Diego, CA)), or M2 (IL-4 20 ng/ml; eBioscience) macrophages. Cells were stimulated for 6 hours prior to RNA isolation. Three independent biological replicate experiments were performed.

### RNA isolation and gene expression

For RNA analysis, Trizol reagent (Life Technologies) was added to single cell suspensions or BMDM culture plates. Total RNA was isolated based on the manufacturer’s protocol, with an additional phase separation using BCP, precipitation with isopropanol (Sigma-Aldrich, St. Louis, MO), and wash of the isolated RNA in 70% ethanol. Then, 1 μg RNA was reverse-transcribed using the high capacity cDNA reverse transcription kit (Life Technologies). Gene expression analysis was performed by using custom Taqman microarray cards seeded with genes reported to be specific to different macrophage phenotypes (Fig. 2) 22. The mixture of 100 ng cDNA sample and Taqman Universal PCR Master Mix (Life Technologies, Grand Island, NY) was loaded on the card. The array was then run under the Custom MicroRNA TaqMan^®^ Array Card (Invitrogen, Carlsbad, CA) default thermal-cycling conditions by ViiA™ 7 Real-Time PCR System (Life Technologies). Expression of genes was normalized to 18S mRNA for each sample.

Real-Time PCR data was analyzed using the comparative Ct (2^ΔΔCT^) method with expression relative to unstimulated BMDMs or same-day SCI vehicle control. SCI data were transformed using the natural logarithm (ln(x)). CT values for “undetermined” genes were imputed and assigned a value of 27 (1 CT value greater than the lowest detected sample). For BMDM gene expression, the “Preprocess Dataset Module” (Genepattern statistical software)^27^ was used to remove inherent technical variability and genes with less than 1 fold change across samples. The “Comparative Marker Selection Suite” (Genepattern)^24^ was then used to compare M1 and M2 stimulated BMDMs and identify genes significantly upregulated by each phenotype.

### Statistical Analyses

Investigators blinded to experimental conditions performed all data acquisition and analysis. Statistical analyses were completed using GraphPad Prism 6.0 (GraphPad Software) or Genepattern^27^. For array analysis of the BMDM gene set, false-positives and family-wise error were controlled using the Family Wise Error Rate (FWER) and setting a conservative significance value at p < 0.01. SCI data were analyzed using one- or two-way ANOVA followed by Dunnett’s test for group comparisons or t-tests when appropriate and results were considered statistically significant at p ≤ 0.05. Grubb’s test (extreme studentized deviate method; http://www.graphpad.com/quickcalcs/grubbs1) was applied to identify and remove aberrant statistical outliers. Two data points were identified as statistical outliers (both were greater >2.5 standard deviations from the mean) and were removed. All data are presented as mean ± SEM unless otherwise noted. Figures were prepared using Adobe Photoshop CS6 (Adobe Systems), Prism 6.0, and Genepattern.

## Additional Information

**How to cite this article**: Gensel, J. C. *et al*. Predictive screening of M1 and M2 macrophages reveals the immunomodulatory effectiveness of post spinal cord injury azithromycin treatment. *Sci. Rep.* **7**, 40144; doi: 10.1038/srep40144 (2017).

**Publisher's note:** Springer Nature remains neutral with regard to jurisdictional claims in published maps and institutional affiliations.

## Supplementary Material

Supplemental Figure S1
