# Supplementary material for: Predictive screening of M1 and M2 macrophages reveals the immunomodulatory effectiveness of post spinal cord injury azithromycin treatment
Source: Sci Rep. 2017 Jan 6;7:40144. doi: 10.1038/srep40144 (PMC5216345; doi:10.1038/srep40144)
Supplement: Supplemental Figure S1 [file srep40144-s1.pdf]

Predictive screening of M1 and M2 macrophages reveals the immunomodulatory effectiveness of post spinal cord injury azithromycin treatment.

Authors: John C. Gensel*, Timothy J Kopper, Bei Zhang, Michael B. Orr, William M. Bailey

Author Affiliation:
Spinal Cord and Brain Injury Research Center,
Department of Physiology,
College of Medicine
University of Kentucky
Lexington, Kentucky 40536

***Correspondence to:**
John C. Gensel
Assistant Professor of Physiology
Spinal Cord and Brain Injury Research Center
B463 Biomed & Biological Science Research Building (BBSRB)
University of Kentucky
741 S. Limestone Street
Lexington, KY 40536-0509

Office: (859) 218-0516
Email: gensel.1@uky.edu

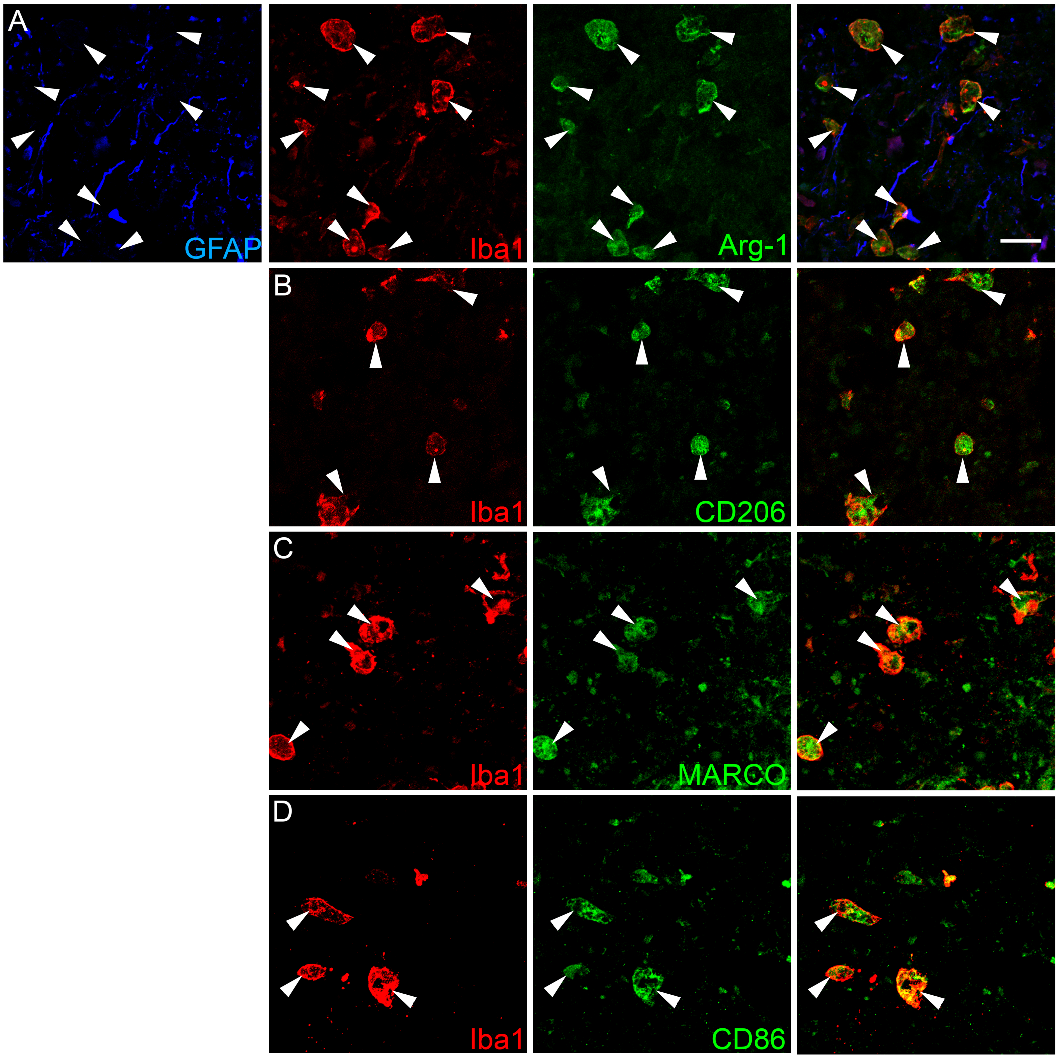

Supplemental Figure S1: Markers of M1 and M2 almost exclusively co-localize with activated macrophages. A) The macrophage marker IBA-1 is specific for macrophages (arrowheads) and does not label other glia (astrocytes-GFAP). A-D) There was no evidence of M2 and M1 markers arginase (Arg-1), CD206, MARCO, and CD86 labeling cells other than macrophages (IBA-1+) in the injured spinal cord. All images are from the lesion epicenter and are representative of both AZM and vehicle at 3 dpi. Cells positive for M2 or M1 markers are also positive for IBA-1 (arrowheads). Similar specificity of markers was observed at 7dpi across both treatment groups (data not shown). Scale bar= 20µm.
